# rs11190870 is not associated with severity of adolescent idiopathic scoliosis in Japanese

**DOI:** 10.1186/1748-7161-10-S1-O3

**Published:** 2015-01-19

**Authors:** Yohei Takahashi, Ikuyo Inaba, Katsuki Kono, Noriaki Kawakami, Koki Uno, Manabu Ito, Shohei Minami, Haruhisa Yanagida, Hiroshi Taneichi, Ikuho Yonezawa, Yoji Ogura, Taichi Tsuji, Teppei Suzuki, Hideki Sudo, Toshiaki Kotani, Kota Watanabe, Yoshiaki Toyama, Morio Matsumoto, Shiro Ikegawa

**Affiliations:** 1 Department of Orthopaedic Surgery, Keio University, Japan; 2 Laboratory of Bone and Joint Diseases, RIKEN Center for Integrative Medical Sciences, Japan; 3 Department of Orthopaedic Surgery, Japanese Red Cross Shizuoka Hospital, Japan; 4 Department of Orthopaedic Surgery, Eiju General Hospital, Japan; 5 Department of Orthopaedic Surgery, Meijo Hospital, Japan; 6 Department of Orthopaedic Surgery, Kobe Medical Center, Japan; 7 Department of Advanced Medicine for Spine and Spinal Cord Disorders, Hokkaido University Graduate School of Medicine, Japan; 8 Department of Orthopaedic Surgery, Seirei Sakura Citizen Hospital, Japan; 9 Department of Orthopaedic Surgery, Fukuoka Children's Hospital, Japan; 10 Department of Orthopaedic Surgery, Dokkyo Medical University School of Medicine, Japan; 11 Department of Orthopaedic Surgery, Juntendo University School of Medicine, Japan

## Objective

We previously conducted a genome-wide association study for AIS susceptibility in Japanese and identified a SNP, rs11190870 near the LBX1 gene on chromosome 10q24.1 that showed significant association. The association is replicated in other populations of different ethnicities. However, it is controversial whether rs11190870 is also associated with severity of the scoliosis curve in AIS. To examine whether rs11190870 is associated with curve severity of AIS in Japanese, we investigated the association using 2,191 (2,068 female and 123 male) subjects.

## Materials and methods

The subjects had been diagnosed with AIS between 10 and 18 years of age. Since AIS may progress until skeletal maturity, we only included subjects with the Risser sign of 4 or 5. The subject's Cobb's angle was the measurement at the last visit after skeletal maturity for non-operated subjects and at the last examination before surgery for operated subjects, respectively. We divided subjects into thoracic and lumber main curve. Because thoracic main curve is a common AIS curve type, we choose subjects with thoracic main curve from skeletal matured female. The association of the Cobb angle of skeletally matured subjects and their genotypes were evaluated. Kruskal-Wallis test was used to compare the Cobb's angle with the different genotypes. rs11190870 was genotyped using the PCR-based Invader assay.

## Results

The association of the Cobb angle of the subjects who reached skeletal maturity and their genotypes were evaluated. By the genotype, subjects were divided into TT, TC, CC groups; 673, 726, 157 in 1556 females, 444, 474, 103 in 1021 females with thoracic main curve and 41, 42, 5 in 88 males, respectively. Average Cobb's angle (S.D.) is 39.0 (15.4), 40.2 (16.4), 37.6 (14.6) in the female group, 40.7 (15.4), 41.7 (16.7), 40.9 (14.2) in the female with thoracic main curve group and 43.6 (16.4), 37.1 (14.0), 30.6 (13.1) in the male group, respectively. P value by Kruskal-Wallis is 0.20 in the female, 0.78 in the female with thoracic main curve and 0.07 in the male groups, respectively.

## Conclusion

No association was found between rs11190870 and curve severity in any subgroup tested, although marginal association was found in the male group. The sample size is so small to conclude. Large-scale replication studies in other ethnic groups would be helpful to clarify the association.

